# The risk for psychiatric disorders in offspring from thrombosis-prone pedigrees in Sweden: a nationwide family study

**DOI:** 10.1016/j.rpth.2025.102692

**Published:** 2025-01-28

**Authors:** Bengt Zöller, Jan Sundquist, Kristina Sundquist, Henrik Ohlsson

**Affiliations:** Department of Clinical Sciences, Malmö, Center for Primary Health Care Research, Lund University, Region Skåne, Malmö, Sweden

**Keywords:** depression, epidemiology, family, mental disorders, venous thromboembolism

## Abstract

**Background:**

Psychiatric disorders have been associated with venous thromboembolism (VTE). However, to our knowledge, no nationwide study has examined the familial association between VTE and psychiatric disorders.

**Objectives:**

We took a pedigree-based approach and examined the risk of psychiatric disorders in offspring from extended pedigrees according to the densities of VTE in pedigrees.

**Methods:**

This was a Swedish national family study. We identified a total of 482,184 Swedish pedigrees from the Swedish Multigeneration Register containing a mean of 14.2 parents, aunts/uncles, grandparents, and cousins of a core full-sibship that we termed the pedigree offspring (*n* = 751,060). We then derived 8 empirical classes of these pedigrees based on the density of cases of VTE. The risk was determined in offspring for psychiatric disorders as a function of VTE density in their pedigrees. Diagnoses of VTE and psychiatric disorders (F00-F69) were determined according to the International Classification of Diseases codes in Swedish registers. All results were Bonferroni corrected.

**Results:**

Higher VTE density, especially for females in pedigrees, was significantly but weakly associated in the offspring with a higher risk of psychiatric disorders. Moreover, VTE density in pedigrees was significantly associated in the offspring with substance abuse disorders, mood (affective) disorders, neurotic, stress-related, and somatoform disorders, behavioral syndromes associated with psychological disturbances and physical factors, personality disorders of adult personality and behavior, depression, and anxiety disorders.

**Conclusion:**

Offspring of pedigrees with a high density of VTE, especially for females, are slightly disadvantaged regarding several psychiatric disorders. VTE shares familial susceptibility, albeit weak, with several psychiatric disorders.

## Essentials

• We determined the risk of psychiatric disorders in thrombosis-prone pedigrees.
• The study design was a Swedish national family study of offspring to extended pedigrees.
• Venous thromboembolism in pedigrees was associated with psychiatric disorders in offspring.
• Venous thromboembolism shares some familial susceptibility with several psychiatric disorders.

## 1 Introduction

Venous thromboembolism (VTE) is a complex disorder where both genetic and acquired factors are important [[1], [2], [3], [4], [5]]. Common risk factors for VTE are old age, obesity, tall body height, malignancy, inflammatory disorders, surgery, trauma, immobilization, pregnancy, oral contraceptives, family history, and genetic predisposition [[1], [2], [3], [4], [5]]. VTE aggregates in families, and several genetic risk factors for VTE have been established [[3], [4], [5], [6], [7], [8], [9], [10], [11], [12]]. Twin, adoption, and family studies indicate that genetic factors contribute to familial transmission of VTE, while familial environmental factors are less important [4,5,[13], [14], [15]]. There is a low spouse correlation of VTE, thus further indicating only a minor nongenetic familial component [14]. Moreover, family studies have shown that the family history of VTE in a first-, second-, and third-degree relative is a risk factor for VTE and its different manifestations [4,5,[13], [14], [15]]. Family history of VTE is a potentially useful clinical and research tool [4,5], and inheritance of VTE could be explained by major genes [13]. In a recent nationwide pedigree-based approach, VTE density in 482,185 Swedish pedigrees was significantly associated in the offspring with different VTE manifestations and several cardiometabolic conditions, ie, obesity, diabetes, gout, varicose veins, arterial embolism, and thrombosis (excluding brain and heart) [16]. However, no significant associations were observed for other cardiometabolic conditions such as hypercholesterolemia, hypertension, coronary heart disease (CHD), myocardial infarction, ischemic stroke, atrial fibrillation, heart failure, cerebral hemorrhage, aortic aneurysm, and peripheral artery disease [16]. Thus, a family history of VTE is specifically related to an increased risk of different VTE manifestations and certain cardiometabolic disorders [16].

Psychiatric disorders are highly prevalent worldwide and have been linked to an increased risk of arterial cardiovascular disorders (CVDs) [17,18]. There is an increasing number of studies reporting an association between different psychiatric disorders and VTE [[19], [20], [21], [22], [23], [24], [25], [26], [27], [28], [29], [30], [31], [32], [33], [34], [35]]. Depression, anxiety disorders, schizophrenia, bipolar disorders, and psychotic disorders have been associated with an increased risk for VTE [[19], [20], [21], [22], [23], [24], [25], [26], [27], [28], [29], [30], [31], [32], [33], [34], [35]]. Several mechanisms for these associations have been suggested, such as the presence of obesity, smoking, systemic and localized inflammation, hypercoagulability, and medication [[36], [37], [38], [39], [40], [41], [42]]. Psychiatric disorders are also linked to an increased risk of arterial CVDs, such as stroke and CHD, and epidemiological studies have documented an association between arterial CVDs and VTE [43]. A bidirectional association has been described with an increased risk of depression after a VTE episode [35,[44], [45], [46]]. Recently, a Mendelian randomization study suggested that depression is causally related to VTE and that genetic factors contribute to the association between depression and VTE [47]. Psychiatric multimorbidity has also been associated with VTE [48]. It has been shown that psychiatric multimorbidity aggregates in families [49], but it has not been reported whether psychiatric disorders and VTE aggregate in the same families.

To the best of our knowledge, whether psychiatric disorders aggregate in families with VTE has not been determined. In the present study, we took a pedigree-based approach according to Kendler et al. [50]. As previously described, starting from a core set of full siblings, we defined pedigrees to include their biological parents, biological uncles and aunts, grandparents, and cousins [16,50]. We divided these extended families into what we called the pedigree (parents, uncles/aunts, grandparents, and cousins) and the core set of full siblings, who we termed the offspring of the pedigree. We then addressed the risk in offspring for different psychiatric disorders as a function of VTE density in their pedigrees.

## 2 Methods

The authors assert that all procedures contributing to this work comply with the ethical standards of the relevant national and institutional committees on human experimentation and with the Helsinki Declaration of 1975, as revised in 2008. We secured approval for this study from the Regional Ethical Review Board in Lund. Data may be obtained from a third party and are not publicly available. We are not allowed to share our data as the data are drawn from registries owned by a third party, ie, the Swedish authorities. The reason behind this is that public availability could potentially compromise patient confidentiality or privacy. Permission to use the data is issued to researchers (if certain conditions are fulfilled) by the National Board of Health and Welfare and by Statistics Sweden. Requests to use the data can be made from the Swedish National Board of Health and Welfare (https://www.socialstyrelsen.se/en/statistics-and-data/statistics/statistical-database/) and Statistics Sweden (https://www.scb.se/en/About-us/contact-us/).

### 2.1 Study design

This nationwide family study utilized several different nationwide Swedish population-based registers, and we linked them using each person’s unique identification number [[51], [52], [53], [54], [55], [56], [57]]. To preserve confidentiality, this identification number was replaced by a serial number created by Statistics Sweden [16,50].

### 2.2 Study pedigrees

The study design has previously been described in detail [16,50,52]. From the Swedish Multigeneration Register [52], we selected all individuals (who form the offspring of our pedigrees) born in Sweden between 1960 and 1970 who still resided in Sweden at age 16 (*N* = 1,693,448) [16]. For each set of offspring (full siblings of one another), we identified parents, grandparents, biological aunts and uncles, cousins, half-siblings, and full siblings. These extended families were divided into what we called *the pedigree* (parents, uncles/aunts, grandparents, and cousins) and the core set of full siblings who, for concision, were termed the *offspring* of the pedigree as previously described [16,50]. The full siblings had to be born outside of the period from 1960 to 1970, as individuals born in that decade constitute the offspring sample. Furthermore, we required that the relative should be alive, residing in Sweden in 1973, and not be born later than 1975. Note that a relative could be included in several different pedigrees, which possibly could inflate the results. However, a total of 59% of the individuals only belonged to 1 pedigree, 23% belonged to 2 pedigrees, and 10% to 3 pedigrees. Thus, only 8% of individuals belonged to more than 3 pedigrees. Rates of VTE in relation to the number of pedigrees an individual belonged to were calculated [16]. Individuals belonging to only 1 pedigree had the highest rates of VTE. VTE rates decreased with the number of pedigrees an individual belonged to. Thus, it is unlikely that the inclusion of patients in several pedigrees inflated the results. For each individual within a pedigree, we identified if they had a lifetime registration of VTE in the National Patient Registry (NPR) or regional primary care registers [16,53,[55], [56], [57]]. VTE was defined using the following International Classification of Diseases (ICD) codes: ICD 10th Revision: I636, I676, I80, I81, I82, I26, O222, O223, O225, O229, O870, O871, O873, O879, O882, O082, and O087; ICD Ninth Revision: 437G, 451, 452, 453, 415B, 416W, 671C, 671D, 671E, 671F, 671X, 639G, and 673C; ICD Eighth Revision: 321, 450, 451, 452, 453, 671, and 673.9 [16]. The diagnosis of VTE in the NPR has been shown to have a validity of 95% [58], whereas the overall validity of the NPR is 87% for all diagnoses [53]. Quality control of 118 patients with VTE in the Malmö Diet and Cancer study cohort [59] was performed. In 106 (90%) cases, the diagnosis was correct [59]. Furthermore, we recorded the number of males/females within each pedigree as well as the number of men/women registered with VTE.

The following comorbidities were defined using the ICD 10th Revision, ICD Ninth Revision, and ICD Eighth Revision according to Supplementary Table S1: obesity, CHD, stroke, cancer, rheumatoid arthritis, inflammatory bowel disorders, diabetes, asthma, chronic obstructive pulmonary disorders, coeliac disease, and gout. Educational status was categorized into a 3-level variable (low [<11 years], mid [12 years], and high [at least 13 years of education]).

### 2.3 Statistical analysis

To stabilize the variance of the estimates for further use, we transformed the rates of males and females with VTE using the Freeman and Tukey arcsine transformation [16,60]. Based on the bivariate transformed rates, we used k-means clustering to partition the pedigrees into a prespecified number of clusters [16,61]. The purpose of using k-means clustering is to define clusters to such an extent that the total intracluster variation, or total within-cluster sum of squares (WSS), is minimized. The total WSS measures the compactness of the clustering, and this should be as small as possible. A common way to determine the number of clusters is the Elbow method [16]. The Elbow method looks at the total WSS as a function of the number of clusters, ie, the smallest number of clusters, which accounts for the largest amount of variation in the data [16]. We chose to work with 8 clusters as, at this number, the curve was close to 90% and clearly began to asymptote [16]. The intraclass correlation (ICC) was determined as a measure of how much the pedigree level explains the total individual variance in VTE [16]. The ICC is used to quantify the degree to which individuals within the same pedigree resemble each other. This is calculated from the variance components from a multilevel model with 8,174,297 individuals nested within the 354,472 pedigrees [16]. We estimated how much of the total variance in VTE could be attributed to the pedigree level and used the latent variable model to calculate the ICC [16]. We then calculated the mean genetic resemblance of pedigree members to the offspring. We first calculated the genetic resemblance between individuals in the pedigree and the proband (0.5 for parents and siblings; 0.25 for half-siblings, grandparents, aunts, and uncles; and 0.125 for cousins). Thereafter, we calculated the mean genetic resemblance within each pedigree. It was important that this did not vary substantially across pedigree classes [16]. If genetic resemblance was higher in the VTE denser pedigrees, this could bias upward the rates of illness in offspring.

Next, we investigated how the offspring of the 8 pedigree clusters (representing different densities of VTE) differed in their risk for several psychiatric variables defined in Table 1. We regressed the mean rates of VTE (across each pedigree cluster) on each of the measured individual variables across all 8 clusters. The corresponding B coefficients are interpreted as the increase/decrease in the variable being studied for each percentage-unit increase in VTE in the pedigree. An interaction term was created to test for an interaction with sex. An analysis was also performed with exclusions of VTE cases among offspring. Stratified analysis was performed according to educational attainment but also after the exclusion of offspring with relevant comorbidities (Supplementary Table S1). We also present regression models between psychiatric diseases in pedigree classes and psychiatric diseases in offspring. To test if these prevalences of psychiatric diseases in pedigrees correspond with the prevalence of psychiatric diseases in offspring, we performed the same model but included mean rates of the psychiatric disorder in the pedigree and its interaction with mean rates of VTE in the cluster. This was a test of whether the effect of psychiatric disorders was different in the different clusters. The *p* values for the interaction terms are presented. *P* values of .05 were Bonferroni corrected for 13 tests among the offspring of the pedigree, *p* = .0038. The statistical analysis was performed using SAS 9.4 (SAS Institute) and R version 3.4.1 (R Core Team, R Foundation for Statistical Computing).

**Table 1:** The 13 studied psychiatric disorders using the International Classification of Diseases in the Swedish National Patient Registry.

Psychiatric disorders	ICD-10	ICD-9	ICD-8
Psychiatric disease	F00-F69	290-305	290-304
Organic, including symptomatic mental disorders	F00-F09	290, 293, 294	290, 292, 293, 294
Substance use disorders	F10-F19	291, 292, 303, 304, 305	291, 303, 304
Schizophrenia and schizotypal and delusional disorders	F20-F29	295, 297, 298, 299	295, 297, 298, 299
Mood (affective) disorders	F30-F39	296	296
Neurotic, stress-related, and somatoform disorders	F40-F48	300	300
Behavioral syndromes associated with psychological disturbances and physical factors	F50-F59	302, 307B, 307E, 307F	302, 30640, 30650
Personality disorders of adult personality and behavior	F60-F69	301	301
Schizophrenia	F20	295	295
Bipolar disorder	F31	296C, 296D, 296E	29610, 29620, 29630, 29688
Depression	F32, F33	296B, 311	29600
Anxiety disorders	F40, F41	300A, 300C	30000, 30020
Personality disorders	F60-F61	301	301

ICD-8/9/10, International Classification of Diseases Eighth/Ninth/10th Revision.

## 3 Results

### 3.1 Pedigrees

We identified a total of 836,657 pedigrees, but to ensure adequate pedigree size, we excluded pedigrees with <7 individuals (*n* = 354,472). We had 482,184 pedigrees for our analyses with a mean (SD) pedigree size of 14.2 (6.5) individuals and 751,060 offspring [16] (Table 2). The results of the k-means clustering for VTE in the pedigrees have previously been published and presented in exact detail [16]. In short, across the 8 pedigree categories, the percentage of individuals affected by VTE increased from 0.5% in class 1 to ≥20.8% in the high-density VTE classes 6 to 8. The mean genetic relationship among members of these pedigrees to the offspring was stable across the classes, ranging narrowly from +0.26 to +0.29 [16].

**Table 2:** Descriptive data of pedigrees, including biological parents, biological uncles and aunts, grandparents, and full cousins to the core set of full siblings.

Descriptive	None	Low female	Low male	Mid female	Mid male	Mid female/male	High female	High male
Class number	1	2	3	4	5	6	7	8
No. of pedigrees	254,216	84,792	62,664	21,005	27,086	21,615	5106	5700
Percentage of total no. of pedigrees	52.7	17.6	13.0	4.4	5.6	4.5	1.1	1.2
No. of members (mean)	14.3	14.6	15.7	12.8	11.9	12.7	10.9	11.4
Mean genetic resemblance	0.27	0.26	0.26	0.28	0.28	0.27	0.29	0.29
Sex (percentage males)	49.5	48.6	52.0	53.7	46.3	50.2	53.8	45.5
Mean year of birth	1944	1944	1945	1943	1942	1942	1941	1941
Mean no. with VTE	0.12	1.25	1.26	2.07	1.64	2.53	3.05	3.17
Percentage with VTE	0.5	9.0	8.3	16.4	13.8	20.8	29.0	28.8
Mean no. of VTE males	0.05	0.12	1.09	0.24	1.51	1.27	0.50	2.43
Mean no. of VTE females	0.07	1.13	0.17	1.83	0.13	1.27	2.55	0.74
No. of VTE cases	30,106	105,863	78,955	43,487	44,483	54,759	15,582	18,071
Percentage of all VTE cases	7.7	27.1	20.2	11.1	11.4	14.0	4.0	4.6
Psychiatric disorder (%)	32.7	33.8	33.4	33.3	33.5	33.6	33.1	34.3
Organic, including symptomatic mental disorders (%)	5.7	5.7	5.4	6.1	6.2	6.1	6.5	6.3
Substance use disorders (%)	7.6	8.0	8.0	8.4	7.5	7.9	8.0	7.6
Schizophrenia and schizotypal and delusional disorders (%)	1.7	1.7	1.7	1.8	1.7	1.7	1.9	1.7
Mood (affective) disorders (%)	12.5	13.3	13.0	12.8	12.9	13.1	13.0	13.6
Neurotic, stress-related, and somatoform disorders (%)	17.2	18.2	17.9	17.2	17.7	17.7	16.7	18.3
Behavioral syndromes associated with psychological disturbances and physical factors (%)	7.7	8.0	7.9	7.9	7.9	8.0	7.8	8.2
Personality disorders of adult personality and behavior (%)	0.9	1.0	0.9	0.9	0.9	0.9	1.0	0.9
Schizophrenia (%)	0.5	0.5	0.5	0.6	0.5	0.5	0.6	0.5
Bipolar disorder (%)	0.8	0.8	0.8	0.8	0.8	0.8	0.7	0.8
Depression (%)	11.9	12.7	12.4	12.3	12.3	12.5	12.4	13.0
Anxiety disorders (%)	9.7	10.3	10.1	9.7	10.0	10.0	9.6	10.3
Personality disorders (%)	0.7	0.7	0.7	0.7	0.6	0.7	0.7	0.7

The terms low, mid, and high describe the distribution of sex of VTE cases in the pedigrees. They are based on the numbers in the 2 rows “Mean no. of VTE males” and “Mean no. of VTE females.”

VTE, venous thromboembolism.

### 3.2 Offspring

We examined an extensive set of measures in the offspring of these 8 pedigree classes (Table 3), which includes the linear regression coefficient in the final column, representing the mean change per percent increase in VTE density in the pedigree. Sibship sizes of the pedigree offspring were typical, with most clusters having around 1.6 offspring. Sex composition was relatively stable at 50.5% to 51.8% of males across the classes.

**Table 3:** Characteristics of the core set of full siblings, ie, pedigree offspring in relation to the pedigree cluster. Regression of the mean rates of venous thromboembolism (across each pedigree cluster) on each of the measured individual variables across all 8 pedigree clusters.

VTE	None %	Low female %	Low male %	Mid female %	Mid male %	Mid female/male %	High female %	High male %	Beta (95% CIs)^a^
Class number	1	2	3	4	5	6	7	8	
No. of subjects	395,397	131,835	97,959	32,963	42,240	33,760	8002	8904	
Percentage of VTE among females	2.0	2.5	2.7	3.4	3.1	4.2	5.0	5.0	
Percentage of VTE among males	1.6	2.3	2.3	2.8	2.8	3.7	5.3	5.1	
Sex (percentage males)	51.3	51.4	51.3	51.2	51.8	51.7	50.8	50.5	
Mean year of birth	1966	1966	1966	1966	1966	1966	1966	1966	
Psychiatric disorder	36.3	37.5	36.9	38.2	36.8	37.7	39.4	36.9	0.078 (0.06; 0.09)^b^
Organic, including symptomatic mental disorders	0.8	0.8	0.8	0.9	0.8	0.8	0.9	1.0	0.002 (−0.00; 0.005)
Substance use disorders	7.9	8.4	8.1	8.6	7.8	8.3	9.2	7.9	0.02 (0.01; 0.03)^b^
Schizophrenia and schizotypal and delusional disorders	1.5	1.5	1.5	1.5	1.4	1.5	1.7	1.4	−0.00 (−0.005; 0.003)
Mood (affective) disorders	16.5	17.0	16.6	17.4	16.5	17.1	17.6	17.1	0.03 (0.02; 0.04)^b^
Neurotic, stress-related, and somatoform disorders	26.2	27.2	26.9	27.9	26.8	27.5	28.3	26.6	0.069 (0.055; 0.08)^b^
Behavioral syndromes associated with psychological disturbances and physical factors	8.5	8.8	8.5	9.1	8.9	8.8	9.7	9.3	0.025 (0.016; 0.03)^b^
Personality disorders of adult personality and behavior	1.5	1.5	1.5	1.6	1.5	1.5	1.9	1.5	0.006 (0.002; 0.01)^b^
Schizophrenia	0.6	0.6	0.6	0.6	0.6	0-7	0.8	0.5	−0.00 (−0.003; 0.002)
Bipolar disorder	1.3	1.2	1.2	1.3	1.2	1.2	1.1	1.1	−0.003 (−0.006; 0.001)
Depression	15.8	16.3	16.0	16.7	15.9	16.5	17.0	16.5	0.035 (0.02; 0.046)^b^
Anxiety disorders	13.9	14.5	14.0	14.9	14.4	14.7	15.5	13.7	0.04 (0.029; 0.05)^b^
Personality disorders	1.1	1.1	1.0	1.2	1.1	1.0	1.3	1.1	0.004 (0.00; 0.007)

The B coefficients are interpreted as the increase/decrease in the variable being studied for each percentage-unit increase in VTE in the pedigree. The terms low, mid, and high describe the distribution of sex of the VTE cases in the pedigrees (Table 2) and not in the core set of full siblings.

VTE, venous thromboembolism.

a Thirteen tests > *P* value for the test of equality between males and females = .0038.

b Significant slope.

Several psychiatric disorders (defined by ICD codes in Table 1) in offspring were significantly associated with VTE density in pedigrees (Table 3). VTE density in pedigrees was associated in the offspring with psychiatric disorders, substance abuse disorders, mood (affective disorders), neurotic, stress-related, and somatoform disorders, behavioral syndromes associated with psychological disturbances and physical factors, personality disorders of adult personality and behavior, depression, and anxiety disorders. No significant associations were observed for schizophrenia and schizotypal and delusional disorders, schizophrenia, bipolar disorder, and personality disorders (Table 3).

Interaction with sex was tested for all psychiatric conditions in offspring (Table 4). There was no significant interaction between sex and the different investigated psychiatric disorders. However, especially female VTE density in pedigrees was associated with psychiatric disorders in offspring (Table 5). Only 2 associations were significant for male VTE density in pedigrees and psychiatric disorders in offspring.

**Table 4:** Interaction with sex was tested, and *P* values were Bonferroni corrected for multiple comparisons.

VTE	*P* value for the interaction term^a^
Psychiatric disorder	.75
Organic, including symptomatic mental disorders	.527
Substance use disorders	.70
Schizophrenia and schizotypal and delusional disorders	.987
Mood (affective) disorders	.45
Neurotic, stress-related, and somatoform disorders	.45
Behavioral syndromes associated with psychological disturbances and physical factors	.79
Personality disorders of adult personality and behavior	.555
Schizophrenia	.875
Bipolar disorder	.367
Depression	.45
Anxiety disorders	.867
Personality disorders	.646

VTE, venous thromboembolism.

a Thirteen tests > *P* value for the test of equality between males and females = .0038.

**Table 5:** Stratified analysis for male and female groups. Regression of the mean rates of venous thromboembolism (across each pedigree cluster) on each of the measured individual variables across male and female groups, respectively.

VTE	Beta (95% CIs)^a^	Beta (95% CIs)^a^
	Male groups	Female groups
Psychiatric disorder	0.04 (0.02; 0.065)^b^	0.12 (0.099; 0.14)^b^
Organic, including symptomatic mental disorders	0.00 (−0.003; 0.005)	0.004 (-0.00; 0.008)
Substance use disorders	0.00 (−0.01; 0.01)	0.048 (0.036; 0.06)^b^
Schizophrenia and schizotypal and delusional disorders	−0.006 (−0.01; −0.00)	0.002 (−0.004; 0.007)
Mood (affective) disorders	0.01 (−0.004; 0.03)	0.049 (0.03; 0.065)^b^
Neurotic, stress-related, and somatoform disorders	0.04 (0.02; 0.06)^b^	0.098 (0.078; 0.117)^b^
Behavioral syndromes associated with psychological disturbances and physical factors	0.02 (0.006; 0.03)^b^	0.03 (0.02; 0.046)^b^
Personality disorders of adult personality and behavior	0.002 (−0.003; 0.008)	0.01 (0.006; 0.016)^b^
Schizophrenia	−0.004 (−0.007; 0.00)	0.00 (−0.003; 0.004)
Bipolar disorder	−0.006 (−0.01; −0.00)	−0.002 (−0.007; 0.003)
Depression	0.018 (0.00; 0.035)	0.05 (0.036; 0.068)^b^
Anxiety disorders	0.015 (−0.002; 0.03)	0.06 (0.047; 0.078)^b^
Personality disorders	0.002 (−0.003; 0.006)	0.008 (0.003; 0.01)^b^

The B coefficients are interpreted as the increase/decrease in the variable being studied for each percentage-unit increase in VTE in the pedigree.

Male groups = none, low male, mid male, and high male; female groups = none, low female, mid female, and high female.

VTE, venous thromboembolism.

a Thirteen tests > *P* value for the test of equality between males and females = .0038.

b Significant slope.

### 3.3 Additional analysis

Table 6 shows a sensitivity analysis excluding offspring with VTE. The strength of the associations decreased, but 7 associations were still significant after exclusions for 7 psychiatric disorders (Table 6). After the exclusion of VTE cases in offspring, psychiatric disorders, substance abuse disorders, mood (affective disorders), neurotic, stress-related, and somatoform disorders, behavioral syndromes associated with psychological disturbances and physical factors, personality disorders of adult personality and behavior, depression, and anxiety disorders were still significantly associated with the density of VTE in the pedigree even after Bonferroni corrections (Table 6). Personality disorders of adult personality and behavior were significant only without the exclusion of personal history of VTE (Tables 3 and 5).

**Table 6:** Exclusion of offspring with venous thromboembolism. The calculations are the same as the main results in Table 3 but with the exclusion of offspring with venous thromboembolism. Regression of the mean rates of venous thromboembolism (across each pedigree cluster) on each of the measured individual variables across all 8 clusters.

VTE	None %	Low female %	Low male %	Mid female %	Mid male %	Mid female/male %	High female %	High male %	Beta (95% CIs)^a^
Class number	1	2	3	4	5	6	7	8	
No. of subjects	388,326	128,671	95,532	31,946	41,004	32,423	7589	8452	
Percentage of VTE among females	0	0	0	0	0	0	0	0	
Percentage of VTE among males	0	0	0	0	0	0	0	0	
Sex (percentage males)	51.4	51.5	51.4	51.4	51.8	51.8	50.8	50.5	
Mean year of birth	1966	1966	1966	1966	1966	1966	1966	1966	
Psychiatric disorder	36.0	37.2	36.7	37.7	36.5	37.2	38.8	36.5	0.069 (0.05; 008)^b^
Organic, including symptomatic mental disorders	0.8	0.8	0.7	0.9	0.8	0.8	0.8	0.9	0.001 (−0.002; 0.004)
Substance use disorders	7.8	8.2	7.9	8.4	7.7	8.0	8.9	7.7	0.017 (0.009; 0.026)^b^
Schizophrenia and schizotypal and delusional disorders	1.5	1.5	1.4	1.4	1.4	1.5	1.6	1.3	−0.002 (−0.006; 0.002)
Mood (affective) disorders	16.4	16.8	16.4	17.1	16.4	16.8	17.2	16.9	0.026 (0.01; 0.038)^b^
Neurotic, stress-related, and somatoform disorders	26.0	27.0	26.6	27.6	26.6	27.2	28.0	26.4	0.06 (0.048; 0.076)^b^
Behavioral syndromes associated with psychological disturbances and physical factors	8.5	8.7	8.4	8.9	8.8	8.7	9.4	9.2	0.02 (0.01; 0.03)^b^
Personality disorders of adult personality and behavior	1.4	1.5	1.4	1.5	1.5	1.4	1.8	1.5	0.004 (0.00; 0.008)
Schizophrenia	0.6	0.6	0.6	0.6	0.6	0.7	0.8	0.5	−0.001 (−0.004; 0.002)
Bipolar disorder	1.2	1.2	1.2	1.2	1.2	1.2	1.1	1.1	−0.003 (−0.007; 0.00)
Depression	15.7	16.2	15.8	16.5	15.7	16.1	16.6	16.2	0.028 (0.017; 0.04)^b^
Anxiety disorders	13.8	14.4	13.9	14.7	14.2	14.5	15.3	13.5	0.03 (0.02; 0.045)^b^
Personality disorders	1.0	1.1	1.0	1.1	1.0	1.0	1.3	1.1	0.002 (−0.00; 0.006)

The B coefficients are interpreted as the increase/decrease in the variable being studied for each percentage-unit increase in VTE in the pedigree. The terms low, mid, and high describe the distribution of sex of the VTE cases in the pedigrees (Table 2) and not in the core set of full siblings, ie, pedigree offspring.

VTE, venous thromboembolism.

a Thirteen tests > *P* value for the test of equality between males and females = .0038.

b Significant slope.

### 3.4 Stratified analysis

Offspring with comorbidities (Supplementary Table S1) were excluded in a stratified analysis (Supplementary Table S2). After Bonferroni correction (*P* = .0038), VTE in the pedigrees was significantly associated with psychiatric disorders, substance use disorders, neurotic, stress-related, and somatoform disorders, and anxiety disorders in offspring (Supplementary Table S2).

In a stratified analysis with the inclusion of only offspring with low educational attainment (<11 years), VTE in the pedigrees were significantly associated with psychiatric disorders, substance use disorders, neurotic, stress-related, and somatoform disorders, behavioral syndromes associated with psychological disturbances and physical factors, depression, and anxiety disorders among offspring (Supplementary Table S3).

In a stratified analysis with the inclusion of only offspring with mideducational attainment (12 years), VTE in the pedigrees was significantly associated with psychiatric disorders, mood (affective) disorders, neurotic, stress-related, and somatoform disorders, and depression among offspring (Supplementary Table S4).

In a stratified analysis with the inclusion of only offspring with high educational attainment (at least 13 years of education), VTE in the pedigrees was significantly associated with psychiatric disorders, neurotic, stress-related, and somatoform disorders, and anxiety disorders among offspring (Supplementary Table S5).

### 3.5 Psychiatric disease in pedigrees and offspring

In Supplementary Table S6, the distribution of psychiatric disorders in the 8 VTE clusters in the pedigrees is shown. To test if these prevalences correspond to the prevalence of psychiatric diseases in offspring, we performed the same model as described above but included mean rates of the psychiatric disorder in the pedigree and its interaction with mean rates of VTE in the cluster. This was a test of whether the effect of psychiatric disorders was different in the different clusters. In the column to the right, we present the *p* value for the interaction term. Only for personality disorders of adult personality and behavior (*p* < .0001), the *p* value was significant after Bonferroni correction. For the other psychiatric diseases, the prevalences in pedigrees corresponded to the prevalences in the offspring.

## 4 Discussion

Using a total of 482,184 pedigrees from the general Swedish population, we examined VTE risk and psychiatric disorders that occurred in offspring from pedigrees with varying rates of VTE. Previously, VTE has been linked to psychiatric disorders at the individual level, even in prospective follow-up studies [[19], [20], [21], [22], [23], [24], [25], [26], [27], [28], [29], [30], [31], [32], [33], [34], [35]]. However, no family studies have been published. This is the first family study showing that several psychiatric diseases aggregate in the same families as VTE, especially in families with female VTE. This suggests the involvement of shared familial factors. These shared factors could be lifestyle-related factors such as smoking, physical activity, diet, and obesity or genetic factors [47]. We confirm a previous study using a pedigree-based method that depression and VTE share biological mechanisms [47]. The differences in absolute numbers are not high and limit the clinical usefulness of our findings. Still, the underlying familial factors that contribute to these differences might be of clinical importance.

We review 5 of our most interesting findings. First, risks for 8 different psychiatric disorders in the pedigree offspring were associated with the density of VTE cases in the pedigree. Thus, several psychiatric disorders share familial susceptibility, albeit weak, with VTE extending the observed link between VTE and psychiatric disorders to even include familial associations [[19], [20], [21], [22], [23], [24], [25], [26], [27], [28], [29], [30], [31], [32], [33], [34], [35]]. Second, offspring of high-density pedigrees demonstrated a significant albeit weak association with 7 psychiatric disorders, even with the exclusion of VTE cases among offspring. This further strengthens the concept of shared familial susceptibility between VTE and several psychiatric disorders. Third, there were no interactions with sex, although especially female VTE density in pedigrees was linked to psychiatric disorders in offspring. Thus, our results are partly dependent on sex. Fourth, no association was observed between family VTE density and schizophrenia, organic, including symptomatic mental disorders (including dementia), or bipolar disorders. Bipolar disorders and schizophrenia have been associated with VTE at the individual level [21,28,31], but the present results argue against a major shared familial susceptibility between VTE and these disorders. A key concept in genetic epidemiology is whether evidence exists for disease aggregation in families. Clustering of disease is necessary, although not sufficient, to infer a shared genetic basis for disease [4,5]. Shared nongenetic risk factors are more likely to be the cause of the link between bipolar and schizophrenia and VTE. Affective disorders and anxiety have been linked to VTE at the individual level [20,26,27,31,34,35]. Psychiatric multimorbidity has also been linked to VTE [48]. The present study with shared familial susceptibility suggests that affective disorders and anxiety might share not only acquired but also genetic factors. The present study is in line with a previous Mendelian randomization study that suggests that genetic variants for depression are involved in VTE pathology [47]. The present study suggests that it is worthwhile also to perform a Mendelian randomization study for VTE and anxiety disorders, especially in females. The question is whether genetic variants involved in the other associated psychiatric disorders are also involved in VTE. A possible approach to determining this is to use genetic risk scores, as in the published Mendelian randomization study, linking depression causally to VTE [47]. It will be important to determine the shared genetic or nongenetic risk factors for VTE and psychiatric disorders. This might lead to the identification of novel risk factors, elucidation of potential mechanistic pathways, and better risk predictions.

Several mechanisms beyond lifestyle-related factors may contribute to the familial association between VTE and psychiatric disorders. For instance, protein components of the complement and coagulation systems have been implicated as potential pathways in psychosis [62]. Concurrent excess activation of coagulation and complement is important in VTE [63], and complement protein levels and complement activation have been associated with future VTE risk [[64], [65], [66]]. Moreover, inflammation, including NLRP3 activation, has been suggested to be important in both psychiatric disorders and VTE [67,68].

### 4.1 Limitations

These results should be interpreted in the context of potential methodological limitations [16,50]. First, our assessments of VTE and psychiatric disorders are based on administrative data derived from medical records. A limitation is that not all psychiatric diagnoses have been validated in the hospital register. Another limitation is that not all psychiatric diagnoses have been validated in the hospital register. However, psychiatric diagnoses such as chronic tic disorder and obsessive-compulsive disorders (86%-96%), personality disorders (55%-92%), schizophrenia, schizophreniform, and schizoaffective disorders (56%-95.5%) usually have high validity [69]. Moreover, we have no information about lifestyle habits such as smoking, exercise, and diet. However, we have done a stratified analysis of educational attainments, which are associated with lifestyle habits [70,71]. Medical comorbidities may affect the results, but there are still many associations after the exclusion of individuals with comorbidities among offspring. The patterns of VTE concentrated in high-risk pedigrees might differ across countries. For instance, factor V Leiden is highly prevalent in Sweden [72]. The causal relationships between the various traits and disorders in these pedigrees are expected to be complex and may involve not only genetics but also acquired and psychosocial factors. Our results are descriptive in nature, and these analyses cannot claim to elucidate causal pathways. Moreover, as multiple analyses were performed, some associations might be spurious. However, we therefore adjusted for multiple comparisons, which may lead to some “true” associations that will not remain significant after Bonferroni correction. Finally, our choice of 8 categories for our pedigrees was based on a recognized method but involved some subjective judgment [16].

## 5 Conclusions

We extend upon previous studies using a different research paradigm in a large, epidemiologically representative national sample and a broad array of psychiatric disorder outcomes. Specifically, we found that offspring of pedigrees with a high density of VTE, especially female VTE density, are at slightly increased risk for different psychiatric disorders. Further genetic studies might be worthwhile, such as Mendelian randomization studies, to establish causal relationships between VTE and different psychiatric disorders. Although weak associations, the familial vulnerability to VTE is broad and impacts several psychiatric disorders. These novel findings may also have implications for efforts in primary prevention and support to patients, but this must be tested prospectively.
