# Supplementary material for: The risk for psychiatric disorders in offspring from thrombosis-prone pedigrees in Sweden: a nationwide family study
Source: Res Pract Thromb Haemost. 2025 Jan 28;9(1):102692. doi: 10.1016/j.rpth.2025.102692 (PMC11908565; doi:10.1016/j.rpth.2025.102692)
Supplement: Supplementary Material [file mmc1.docx]

**Supplementary Tables S1-S6**

| **Supplementary Table S1.** International Classification of Diseases (ICD) codes for comorbidities. | | | |
| --- | --- | --- | --- |
|  | ICD-10 | ICD-9 | ICD-8 |
| Obesity | E66 | 278 | 277 |
| Coronary heart disease (CHD) | I20-I25 | 410-414 | 410-414 |
| Stroke | I60-I69 | 430-438 | 430-438 |
| Cancer | C00-C99 | 140-208 | 140-209 |
| Rheumatoid arthritis | M05-M06 | 714 (not 714D, 714E, 714X) | 7121, 7123 |
| Inflammatory bowel disease (IBD) | K50, K51 | 555, 556 | 56300, 56310 |
| Diabetes | E10-E14 | 250 | 250 |
| Asthma | J45-J46 | 493 | 493 |
| Chronic obstructive pulmonary disease (COPD) | J40-J44 | 490-492 | 490-492 |
| Coeliac disease | K900 | 579A | 26900, 26998 |
| Gout | M10 | 274 | 274 |

| **Supplementary Table S2.** Stratified analysis of the core set of full siblings i.e. pedigree offspring with exclusion of comorbidities. The terms low, mid, and high describe the distribution of sex of the venous thromboembolism (VTE) cases in the pedigrees in main Table 2 and not in the core set of full siblings. The same calculations as the main results in main Table 3 but with exclusion of relative offspring with venous thromboembolism (VTE), obesity, coronary heart disease (CHD), stroke, cancer, rheumatoid arthritis, inflammatory bowel disease (IBD), diabetes, asthma, chronic obstructive pulmonary disease (COPD), coeliac disease, and gout. | | | | | | | | | | | | | | | | |  |  |
| --- | --- | --- | --- | --- | --- | --- | --- | --- | --- | --- | --- | --- | --- | --- | --- | --- | --- | --- |
| VTE | None | Low Female | | Low Male | Mid Female | | Mid Male | Mid Female/Male | | | High Female | | | High Male | | Beta (95% CIs)* |  |  |
| Class Number | 1 | 2 | | 3 | 4 | | 5 | 6 | | | 7 | | | 8 | |  |  |  |
| Number of subjects | 269,154 | 87,664 | | 65,357 | 21,472 | | 28,306 | 21,846 | | | 4,965 | | | 5,791 | |  |  |  |
| Percentage of VTE among females | 0% | 0% | | 0% | 0% | | 0% | 0% | | | 0% | | | 0% | |  |  |  |
| Percentage of VTE among males | 0% | 0% | | 0% | 0% | | 0% | 0% | | | 0% | | | 0% | |  |  |  |
| Sex (percentage males) | 53.8% | 54.0% | | 53.9% | 54.4% | | 54.3% | 54.6% | | | 54.4% | | | 53.1% | |  |  |  |
| Mean year of birth | 1966 | 1966 | | 1966 | 1966 | | 1966 | 1966 | | | 1966 | | | 1966 | |  |  |  |
| Psychiatric Disorder | 30.7% | 31.6% | | 31.2% | 31.8% | | 31.2% | 31.5% | | | 32.7% | | | 31.1% | | 0.054 (0.036; 0.072)** |  |  |
| Organic, including symptomatic, mental disorders | 0.5% | 0.5% | | 0.5% | 0.6% | | 0.5% | 0.4% | | | 0.5% | | | 0.7% | | 0.001 (-0.001; 0.004) |  |  |
| Substance use disorders | 5.8% | 6.1% | | 5.9% | 6.3% | | 5.8% | 5.8% | | | 6.7% | | | 5.6% | | 0.013 (0.004, 0.022)** |  |  |
| Schizophrenia and schizotypal and delusional disorders | 1.3% | 1.2% | | 1.1% | 1.2% | | 1.2% | 1.2% | | | 1.5% | | | 1.1% | | -0.003 (-0.007; 0.002) |  |  |
| Mood [affective] disorders | 13.1% | 13.3% | | 13.2% | 13.2% | | 13.0% | 13.3% | | | 13.9% | | | 13.4% | | 0.014 (0.001; 0.027) |  |  |
| neurotic, stress related, and somatoform disorders | 22.0% | 22.7% | | 22.4% | 22.9% | | 22.4% | 22.7% | | | 23.4% | | | 22.3% | | 0.047 (0.031; 0.063)** |  |  |
| Behavioral syndromes associated with psychological disturbances and physical factors | 6.8% | 6.8% | | 6.7% | 7.0% | | 6.9% | 6.8% | | | 7.2% | | | 7.3% | | 0.009 (-0.000; 0.019) |  |  |
| Personality disorders of adult personality and behavior | 1.1% | 1.1% | | 1.1% | 1.1% | | 1.1% | 1.0% | | | 1.4% | | | 1.3% | | 0.004 (-0.000; 0.008) |  |  |
| Schizophrenia | 0.5% | 0.5% | | 0.5% | 0.5% | | 0.5% | 0.6% | | | 0.6% | | | 0.3% | | -0.001 (-0.004; 0.001) |  |  |
| Bipolar disorder | 0.9% | 0.9% | | 0.9% | 0.9% | | 0.9% | 0.8% | | | 0.6% | | | 0.9% | | -0.005 (-0.009; -0.001) |  |  |
| Depression | 12.5% | 12.8% | | 12.7% | 12.6% | | 12.5% | 12.7% | | | 13.3% | | | 13.0% | | 0.016 (0.003; 0.029) |  |  |
| Anxiety disorders | 11.0% | 11.5% | | 11.1% | 11.5% | | 11.3% | 11.6% | | | 12.2% | | | 10.5% | | 0.025 (0.013; 0.037)** |  |  |
| Personality disorders | 0.8% | 0.8% | | 0.8% | 0.8% | | 0.8% | 0.7% | | | 1.0% | | | 1.0% | | 0.002 (-0.002; 0.005) |  |  |
| N=number; VTE=venous thromboembolism, *13 test - >P-value for test of equality between male and females = 0.0038 - ** = Significant slope | | | | | | | | | | | | | | | | |  |  |
| **Supplementary Table S3.** Stratified analysis of the core set of full siblings i.e. pedigree offspring with inclusion of offspring with low education (<11 years). The terms low, mid, and high describe the distribution of sex of the venous thromboembolism (VTE) cases in the pedigrees in Table 2 and not in the core set of full siblings. The same calculations as the main results in main Table 3 but with inclusion only of offspring with low education (<11 years). | | | | | | | | | | | | | | | | | |  |
| VTE | None | Low Female | Low Male | | Mid Female | Mid Male | | Mid Female/Male | | High Female | | | High Male | | | Beta (95% CIs)* | |  |
| Class Number | 1 | 2 | 3 | | 4 | 5 | | 6 | | 7 | | | 8 | | |  | |  |
| Number of subjects | 180,334 | 62,235 | 45,872 | | 15,891 | 19,269 | | 15,745 | | 3,850 | | | 4,033 | | |  | |  |
| Percentage of VTE among females | 2.5% | 3.0% | 3.3% | | 4.1% | 4.0% | | 5.0% | | 6.1% | | | 6.2% | | |  | |  |
| Percentage of VTE among males | 1.8% | 2.5% | 2.5% | | 3.2% | 3.0% | | 4.3% | | 6.0% | | | 5.6% | | |  | |  |
| Sex (percentage males) | 60.6% | 60.4% | 60.9% | | 59.6% | 60.9% | | 60.6% | | 59.8% | | | 59.8% | | |  | |  |
| Mean year of birth | 1965 | 1965 | 1965 | | 1965 | 1965 | | 1965 | | 1965 | | | 1965 | | |  | |  |
| Psychiatric Disorder | 38.9% | 40.1% | 39.5% | | 40.5% | 39.4% | | 40.3% | | 41.7% | | | 39.7% | | | 0.072 (0.050; 0094)** | |  |
| Organic, including symptomatic, mental disorders | 1.1% | 1.2% | 1.0% | | 1.2% | 1.1% | | 1.1% | | 1.2% | | | 1.3% | | | 0.003 (-0.002; 0.008) | |  |
| Substance use disorders | 12.0% | 12.7% | 11.9% | | 12.6% | 11.8% | | 12.4% | | 13.8% | | | 11.8% | | | 0.022 (0.007, 0.037)** | |  |
| Schizophrenia and schizotypal and delusional disorders | 2.1% | 2.0% | 2.0% | | 2.0% | 1.9% | | 2.1% | | 2.3% | | | 2.0% | | | -0.001 (-0.008; 0.006) | |  |
| Mood [affective] disorders | 17.6% | 18.0% | 17.5% | | 18.6% | 17.5% | | 18.1% | | 18.4% | | | 18.3% | | | 0.027 (0.009; 0.044) | |  |
| neurotic, stress related, and somatoform disorders | 26.7% | 27.9% | 27.4% | | 28.3% | 27.5% | | 28.2% | | 29.0% | | | 27.1% | | | 0.069 (0.049; 0.090)** | |  |
| Behavioral syndromes associated with psychological disturbances and physical factors | 9.0% | 9.2% | 9.1% | | 9.5% | 9.7% | | 9.5% | | 10.5% | | | 10.5% | | | 0.033 (0.020; 0.046)** | |  |
| Personality disorders of adult personality and behavior | 2.1% | 2.2% | 2.1% | | 2.2% | 2.3% | | 2.2% | | 2.5% | | | 2.2% | | | 0.007 (0.000; 0.014) | |  |
| Schizophrenia | 0.9% | 0.9% | 0.9% | | 0.9% | 0.9% | | 1.0% | | 1.1% | | | 0.9% | | | 0.001 (-0.003; 0.006) | |  |
| Bipolar disorder | 1.4% | 1.3% | 1.3% | | 1.4% | 1.3% | | 1.4% | | 1.2% | | | 1.3% | | | -0.004 (-0.009; 0.002) | |  |
| Depression | 16.8% | 17.3% | 16.8% | | 17.9% | 16.7% | | 17.3% | | 17.8% | | | 17.7% | | | 0.030 (0.010; 0.048)** | |  |
| Anxiety disorders | 15.5% | 16.2% | 15.6% | | 16.2% | 15.9% | | 16.3% | | 17.3% | | | 15.1% | | | 0.037 (0.020; 0.053)** | |  |
| Personality disorders | 1.6% | 1.6% | 1.5% | | 1.7% | 1.7% | | 1.5% | | 1.9% | | | 1.6% | | | 0.004 (-0.001; 0.010) | |  |
| N=number; VTE=venous thromboembolism, *13 test - >P-value for test of equality between male and females = 0.0038 - ** = Significant slope | | | | | | | | | | | | | | | | | |  |
| **Supplementary Table S4.** Stratified analysis of pedigree offspring (i.e. the core set of full siblings) with mid education (12 years). The terms low, mid, and high describe the distribution of sex of the venous thromboembolism (VTE) cases in the pedigrees in Table 2 and not in the core set of full siblings. The same calculations as the main results in main Table 3 but with inclusion of only offspring mid education (12 years). | | | | | | | | | | | | | | | | | | |
| VTE | | None | Low Female | Low Male | | Mid Female | Mid Male | | Mid Female/Male | High Female | | | High Male | | | Beta (95% CIs)* | | |
| Class Number | | 1 | 2 | 3 | | 4 | 5 | | 6 | 7 | | | 8 | | |  | | |
| Number of subjects | | 124,779 | 40,979 | 30,858 | | 10,261 | 13,512 | | 10,638 | 2,539 | | | 2,873 | | |  | | |
| Percentage of VTE among females | | 1.9% | 2.4% | 2.4% | | 3.0% | 2.7% | | 3.9% | 4.9% | | | 4.5% | | |  | | |
| Percentage of VTE among males | | 1.5% | 2.1% | 2.1% | | 2.3% | 2.5% | | 3.1% | 4.0% | | | 5.3% | | |  | | |
| Sex (percentage males) | | 44.8% | 44.8% | 44.2% | | 44.2% | 45.4% | | 45.5% | 42.5% | | | 45.0% | | |  | | |
| Mean year of birth | | 1966 | 1966 | 1966 | | 1966 | 1966 | | 1966 | 1966 | | | 1966 | | |  | | |
| Psychiatric Disorder | | 35.6% | 36.3% | 35.9% | | 37.0% | 36.2% | | 36.7% | 39.1% | | | 35.8% | | | 0.060 (0.034; 0087)** | | |
| Organic, including symptomatic, mental disorders | | 0.6% | 0.6% | 0.6% | | 0.7% | 0.6% | | 0.7% | 0.6% | | | 0.7% | | | -0.000 (-0.004; 0.004) | | |
| Substance use disorders | | 5.5% | 5.7% | 5.6% | | 5.7% | 5.4% | | 5.5% | 6.0% | | | 5.5% | | | 0.007 (-0.007, 0.020) | | |
| Schizophrenia and schizotypal and delusional disorders | | 1.2% | 1.2% | 1.1% | | 1.1% | 1.2% | | 1.2% | 1.3% | | | 0.9% | | | -0.001 (-0.007; 0.005) | | |
| Mood [affective] disorders | | 16.5% | 16.9% | 16.6% | | 16.9% | 16.7% | | 17.6% | 18.0% | | | 16.7% | | | 0.031 (0.010; 0.052)** | | |
| neurotic, stress related, and somatoform disorders | | 26.8% | 27.4% | 27.2% | | 28.2% | 27.3% | | 27.6% | 29.1% | | | 27.1% | | | 0.051 (0.027; 0.076)** | | |
| Behavioral syndromes associated with psychological disturbances and physical factors | | 8.4% | 8.6% | 8.2% | | 8.8% | 8.4% | | 8.5% | 9.2% | | | 8.5% | | | 0.011 (-0.004; 0.027) | | |
| Personality disorders of adult personality and behavior | | 1.1% | 1.1% | 1.0% | | 1.0% | 1.1% | | 1.1% | 1.5% | | | 0.8% | | | 0.000 (-0.005; 0.006) | | |
| Schizophrenia | | 0.5% | 0.4% | 0.4% | | 0.4% | 0.4% | | 0.5% | 0.6% | | | 0.2% | | | -0.003 (-0.007; 0.001) | | |
| Bipolar disorder | | 1.2% | 1.3% | 1.1% | | 1.3% | 1.3% | | 1.3% | 0.9% | | | 0.9% | | | -0.004 (-0.009; 0.003) | | |
| Depression | | 15.9% | 16.2% | 16.0% | | 16.3% | 16.0% | | 17.0% | 17.5% | | | 16.2% | | | 0.033 (0.013; 0.054)** | | |
| Anxiety disorders | | 13.6% | 13.9% | 13.5% | | 14.4% | 14.2% | | 14.2% | 14.9% | | | 13.0% | | | 0.025 (0.006; 0.044) | | |
| Personality disorders | | 0.7% | 0.8% | 0.7% | | 0.7% | 0.7% | | 0.7% | 0.9% | | | 0.7% | | | 0.001 (-0.001; 0.005) | | |
| N=number; VTE=venous thromboembolism, *13 test - >P-value for test of equality between male and females = 0.0038 - ** = Significant slope | | | | | | | | | | | | | | | | | | |

| **Supplementary Table S5.** Stratified analysis of offspring with high education (at least 13 years of education). The core set of full siblings i.e. pedigree offspring. The terms low, mid, and high describe the distribution of sex of the venous thromboembolism (VTE) cases in the pedigrees in Table 2 and not in the core set of full siblings. The same calculations as the main results in main Table 3 but with inclusion only of offspring with high education (at least 13 years of education).. | | | | | | | | | |
| --- | --- | --- | --- | --- | --- | --- | --- | --- | --- |
| VTE | None | Low Female | Low Male | Mid Female | Mid Male | Mid Female/Male | High Female | High Male | Beta (95% CIs)* |
| Class Number | 1 | 2 | 3 | 4 | 5 | 6 | 7 | 8 |  |
| Number of subjects | 83,802 | 26,866 | 19,921 | 6,392 | 8,785 | 6,939 | 1,517 | 1,891 |  |
| Percentage of VTE among females | 1.4% | 1.9% | 2.1% | 2.8% | 2.5% | 3.5% | 3.3% | 4.0% |  |
| Percentage of VTE among males | 1.2% | 2.0% | 2.1% | 2.4% | 2.7% | 3.2% | 5.3% | 3.4% |  |
| Sex (percentage males) | 40.6% | 404% | 40.1% | 41.3% | 41.2% | 40.5% | 41.3% | 39.3% |  |
| Mean year of birth | 1966 | 1966 | 1966 | 1966 | 1966 | 1966 | 1966 | 1966 |  |
| Psychiatric Disorder | 34.0% | 35.1% | 34.5% | 36.2% | 34.5% | 35.2% | 35.5% | 34.1% | 0.065 (0.033; 0098)** |
| Organic, including symptomatic, mental disorders | 0.5% | 0.5% | 0.5% | 0.5% | 0.6% | 0.4% | 0.5% | 0.5% | 0.000 (-0.004; 0.006) |
| Substance use disorders | 3.2% | 3.2% | 3.4% | 3.7% | 3.2% | 3.6% | 3.4% | 3.4% | 0.013 (0.000, 0.025) |
| Schizophrenia and schizotypal and delusional disorders | 0.9% | 0.8% | 0.8% | 1.0% | 0.6% | 0.6% | 1.0% | 0.7% | -0.005 (-0.011; 0.002) |
| Mood [affective] disorders | 15.3% | 15.8% | 15.6% | 16.3% | 15.4% | 15.2% | 15.8% | 16.2% | 0.021 (-0.005; 0.046) |
| neurotic, stress related, and somatoform disorders | 26.0% | 26.9% | 26.6% | 28.2% | 26.5% | 27.5% | 26.6% | 25.9% | 0.059 (0.029; 0.089)** |
| Behavioral syndromes associated with psychological disturbances and physical factors | 8.3% | 8.5% | 8.1% | 9.2% | 8.4% | 8.3% | 8.9% | 8.7% | 0.017 (-0.002; 0.037) |
| Personality disorders of adult personality and behavior | 0.7% | 0.7% | 0.7% | 1.0% | 0.6% | 0.7% | 1.1% | 1.0% | 0.004 (-0.002; 0.010) |
| Schizophrenia | 0.2% | 0.2% | 0.2% | 0.2% | 0.2% | 0.2% | 0.3% | 0.1% | -0.002 (-0.006; 0.001) |
| Bipolar disorder | 1.1% | 1.0% | 1.1% | 1.1% | 0.9% | 0.9% | 1.4% | 1.1% | -0.002 (-0.009; 0.005) |
| Depression | 14.7% | 15.2% | 15.0% | 15.6% | 14.9% | 14.8% | 15.2% | 15.4% | 0.022 (-0.003; 0.047) |
| Anxiety disorders | 12.0% | 12.5% | 12.3% | 13.3% | 12.6% | 12.9% | 13.0% | 12.2% | 0.041 (0.019; 0.064)** |
| Personality disorders | 0.5% | 0.5% | 0.4% | 0.6% | 0.5% | 0.3% | 0.6% | 0.8% | 0.000 (-0.004; 0.005) |
| N=number; VTE=venous thromboembolism, *13 test - >P-value for test of equality between male and females = 0.0038 - ** = Significant slope | | | | | | | | | |

| **Supplementary Table S6.** Distribution of psychiatric disorders in the eight VTE clusters in the pedigrees. To test if these VTE prevalences correspond with the prevalence of psychiatric diseases in offsprings we performed the same model as described in the methods but included mean rates of the psychiatric disorder in the pedigree and its interaction with mean rates of VTE in the cluster. This was a test of if the effect of psychiatric disorder was different in the different clusters. In the column to the right, we present the p-value for the interaction term. | | | | | | | | | |
| --- | --- | --- | --- | --- | --- | --- | --- | --- | --- |
| VTE | None | Low Female | Low Male | Mid Female | Mid Male | Mid Female/Male | High Female | High Male | P-value interaction |
| Class Number | 1 | 2 | 3 | 4 | 5 | 6 | 7 | 8 |  |
| Psychiatric Disorder | 32.7% | 33.8% | 33.4% | 33.3% | 33.5% | 33.6% | 33.1% | 34.3% | 0.1887 |
| Organic, including symptomatic, mental disorders | 5.7% | 5.7% | 5.4% | 6.1% | 6.2% | 6.1% | 6.5% | 6.3% | 0.589 |
| Substance use disorders | 7.6% | 8.0% | 8.0% | 8.4% | 7.5% | 7.9% | 8.0% | 7.6% | 0.584 |
| Schizophrenia and schizotypal and delusional disorders | 1.7% | 1.7% | 1.7% | 1.8% | 1.7% | 1.7% | 1.9% | 1.7% | 0.651 |
| Mood [affective] disorders | 12.5% | 13.3% | 13.0% | 12.8% | 12.9% | 13.1% | 13.0% | 13.6% | 0.085 |
| neurotic, stress related, and somatoform disorders | 17.2% | 18.2% | 17.9% | 17.2% | 17.7% | 17.7% | 16.7% | 18.3% | 0.729 |
| Behavioral syndromes associated with psychological disturbances and physical factors | 7.7% | 8.0% | 7.9% | 7.9% | 7.9% | 8.0% | 7.8% | 8.2% | 0.048 |
| Personality disorders of adult personality and behavior | 0.9% | 1.0% | 0.9% | 0.9% | 0.9% | 0.9% | 1.0% | 0.9% | <0.0001** |
| Schizophrenia | 0.5% | 0.5% | 0.5% | 0.6% | 0.5% | 0.5% | 0.6% | 0.5% | 0.019 |
| Bipolar disorder | 0.8% | 0.8% | 0.8% | 0.8% | 0.8% | 0.8% | 0.7% | 0.8% | 0.947 |
| Depression | 11.9% | 12.7% | 12.4% | 12.3% | 12.3% | 12.5% | 12.4% | 14.0% | 0.080 |
| Anxiety disorders | 9.7% | 10.3% | 10.1% | 9.7% | 10.0% | 10.0% | 9.6% | 10.3% | 0.545 |
| Personality disorders | 0.7% | 0.7% | 0.7% | 0.7% | 0.6% | 0.7% | 0.7% | 0.7% | 0.285 |
| N=number; VTE=venous thromboembolism, *13 test - >P-value for test of equality between male and females = 0.0038 - ** = Significant slope | | | | | | | | | |
